# Low-Density Lipoprotein Receptor-Related Protein 1 (LRP1) Regulates the Stability and Function of GluA1 α-Amino-3-Hydroxy-5-Methyl-4-Isoxazole Propionic Acid (AMPA) Receptor in Neurons

**DOI:** 10.1371/journal.pone.0113237

**Published:** 2014-12-12

**Authors:** Ming Gan, Peizhou Jiang, Pamela McLean, Takahisa Kanekiyo, Guojun Bu

**Affiliations:** 1 Department of Neuroscience, Mayo Clinic, Jacksonville, Florida, United States of America; 2 Fujian Provincial Key Laboratory of Neurodegenerative Disease and Aging Research, Institute of Neuroscience, College of Medicine, Xiamen University, Xiamen, China; Louisiana State University Health Sciences Center, United States of America

## Abstract

The low-density lipoprotein receptor-related protein 1 (LRP1) is a multifunctional endocytic receptor abundantly expressed in neurons. Increasing evidence demonstrates that LRP1 regulates synaptic integrity and function at the post synapses, at least partially by regulating glutamate receptors. The α-amino-3-hydroxy-5-methyl-4-isoxazole propionic acid receptors (AMPARs) are critical ionotropic glutamate receptors consisting of homotetramer or heterotetramer of GluA1-4 subunits and play an essential role in synaptic transmission and synaptic plasticity. Our previous work has shown that neuronal deletion of the *Lrp1* gene in mice leads to decreased level of GluA1 and reduced long-term potentiation. To understand the underlying mechanism, we investigated the cellular and functional consequences of LRP1 deletion in primary neurons. Here, we show that LRP1 interacts with and regulates the cellular distribution and turnover of GluA1. LRP1 knockdown in mouse primary neurons led to accelerated turnover and decreased cell surface distribution of GluA1, which correspond to decreased phosphorylation of GluA1 at S845 and S831 sites. Decreased LRP1 expression also attenuated AMPA-evoked calcium influx and reduced GluA1-regulated neurite outgrowth and filopodia density. Our results reveal a novel mechanism by which LRP1 controls synaptic integrity and function, specifically by regulating GluA1 trafficking, phosphorylation and turnover. They further demonstrate that LRP1-GluA1 pathway may hold promises as a therapeutic target for restoring synaptic functions in neurodegenerative diseases.

## Introduction

The low-density lipoprotein receptor-related protein 1 (LRP1) is a large endocytic receptor abundantly expressed in various brain cell types, including neurons and glial cells in brain parenchyma, and smooth muscle cells and pericytes in cerebrovasculature, where it mediates cellular uptake of diverse ligands including apolipoprotein E (apoE), α2-macroglobulin, and tissue plasminogen activator (tPA) [1], [2], [3]. LRP1 is a highly efficient transport receptor with a rapid endocytosis rate and signal-mediated recycling by interacting with multiple adaptor proteins through several tyrosine-based motifs in its cytoplasmic tail region [4], [5]. Furthermore, LRP1 also regulates signal transduction by coupling with other cell-surface signalling receptors including the platelet-derived growth factor receptor (PDGFR) [6] and the leptin receptor [7]. In neurons, LRP1 is predominantly expressed in the postsynaptic region [8] and the cell body [9], where it regulates lipid transport [10] and the metabolism of amyloid-β (Aβ) peptides [11], [12] whose accumulation is considered central to the pathogenesis of Alzheimer's disease (AD). LRP1 is known to form a complex with N-methyl-d-aspartate receptors (NMDARs) through the multivalent scaffold protein, postsynaptic density protein 95 (PSD95) [8], which modulates synaptic transmission and synaptic plasticity [13], [14], [15].

In addition to NMDARs, another ionotropic glutamate receptor termed α-amino-3-hydroxy-5-methyl-4-isoxazolepropionic acid receptors (AMPARs), consisting of homotetramer or heterotetramer proteins formed by GluA1-4 subunits [16], [17], also critically regulates long-term potentiation (LTP) and long-term depression (LTD) through the phosphorylation and de-phosphorylation of its C-terminal domain [18]. AMPARs rapidly traffic between membrane compartments, where they can be endocytosed and sorted for degradation pathways or for recycling back to the plasma membrane during LTP and LTD [19]. AMPARs also regulate dendrite complexity and spine motility in neurons [20], and contribute to synaptic plasticity and formation through their redistribution to synaptic membranes [21], [22], [23]. Despite the fact that LRP1 is a component of the postsynaptic protein complexes and our recent work showing that neuronal conditional knockout of the *Lrp1* gene leads to decreased level of GluA1 [10], it is not clear how LRP1 regulates AMPARs' expression and function. Thus in this study, we focused on addressing the interaction and functional impacts between LRP1 and the AMPAR subunit GluA1 using mouse primary cortical neurons. Here, we demonstrate that LRP1 controls the cellular distribution, turnover and phosphorylation of GluA1, which in turn influences calcium influx, neurite outgrowth and filopodia formation in neurons.

## Materials and Methods

### Ethics statement

The care and treatments of animals were carried out in strict accordance with the recommendations in the Guide for the Care and Use of Laboratory Animals of the National Institutes of Health. The protocol was approved by the Mayo Clinic Institutional Animal Care and Use Committee (Protocol number A30010). Mice were terminally anesthetized with sodium pentobarbital, and all efforts were made to minimize suffering.

### Plasmids and lentivirus preparation

Lentiviral plasmid CS-Mm02851-Lv206 for expression of GluA1 was purchased from Genecopoeia (Rockville, MD). Lentiviral plasmid carrying shRNA for LRP1 knockdown and non-target (NT) scrambled shRNA as control were purchased from Sigma-Aldrich (St. Louis, MO). Lentiviruses were generated by plasmid transfection with helper plasmids in HEK293FT cells. The media were collected and concentrated by Lentivirus Concentration & Purification Kit (Cell Biolabs, San Diego, CA) after 48 hour transfection, The genomic titer of each virus was determined by qRT-PCR Titration Kit (Cell Biolabs) and Q-PCR using the ABI 7900 (Applied Biosystems, Foster City, CA). The purified viruses with titers ranging from 1×10^9^ to 1×10^11^ viral particles/mL were used.

### Primary neuronal culture

Embryonic primary cortical neurons were prepared from E17 of C57BL/6 wild-type mice, which were from the Jackson Lab (Bar Harbor, Maine). The cortices from the embryos were dissociated with 100 U/ml papain (Fisher, Pittsburgh, PA) and plated at 1×10^6^ cells on poly-D- lysine (Merck, Darmstadt, Germany) coated 6-well plate for biochemical analysis, or 4×10^4^ per well on coverslips in 24-well plate for neurite outgrowth and filopodia analysis, or 2×10^5^ per well in 24-well plate for intracellular Ca^2+^ assay. Cell cultures were maintained at 37°C in a humidified incubator with 5% CO_2_/95% air, and neurons were used for experiments on day 8 *in vitro* (DIV). In some experiments, primary neurons (DIV7) were infected with lentivirus carrying GluA1 plasmid for 24 h, and secondly infected with lentivirus carrying control NT-shRNA or LRP1-shRNA to knockdown LRP1 for 4 days.

### Cell viability

The cell viability was determined by MTT assay with the Cell Proliferation Kit (Roche, Palo Alto, USA). Briefly, neurons were incubated with MTT labeling reagent at a final concentration of 0.5 mg/ml for 4 hours at 37°C. Then, the formazan crystals were dissolved in solubilization solution and incubated overnight at 37°C. The absorbance was quantified at 550 nm and the reference was measured at 690 nm.

### Western blot

Cells were washed and scraped in cold PBS, then lysed in PBS buffer containing 1% Triton-X 100, 1 mM phenylmethylsulfonyl fluoride, and protease inhibitor mixture (Roche). Protein concentrations were determined using a protein assay kit (Bio-Rad, Hercules, CA). Equal amounts of extract from each sample were separated by SDS-PAGE and transferred to the polyvinylidene difluoride (PVDF) membrane, then immunoblotted using primary antibodies. The primary antibodies used were: anti-GluA1 (1∶300, Millipore), anti-GluA2/3 (1∶500, Millipore), anti-pS831-GluA1 (1∶500, Epitomics, Burlingame, CA), anti-pS845-GluA1 (1∶500, Epitomics), anti-LRP1 (1∶2000, made in house) ([12]), anti-PSD95 (1∶1000, Cell Signaling, Danvers, MA) and anti-β actin (1∶10, 000, Sigma-Aldrich). Image acquisition and data quantification were performed on the Odyssey Imaging system (Li-Cor, Lincoln, NE).

### Quantitative real-time PCR

Total RNAs isolated from cells using RNeasy Mini Kit (Qiagen, Valencia, CA, USA) were reverse-transcribed with First-Strand Synthesis System (Invitrogen, Carlsbad, CA). The reaction mix was subjected to Applied Biosystems 7900HT Fast Real-Time PCR System to detect expression levels of GluA1 and PSD95 using mouse quantitative PCR primers specific for corresponding genes. The set of actin primers (Qiagen) was used as an internal control for each specific gene amplification. The primers were as follows: mouse GluA1-F (5′-CGA AGC GGA TGA AGG GTT T-3′), mouse GluA1-R (5′-GGA AGT CCT GGC TGA CCA C-3′); mouse PSD95-F (5′-CGA GGA TGC CGT GGC AGC C-3′), mouse PSD95-R (5′-CAT GGC TGT GGG GTA GTC AGT GCC-3′).

### Protein turnover assays

Neurons were cultured with 100 µg/ml of cycloheximide (CHX, Sigma-Aldrich) to inhibit further protein synthesis. Levels of GluA1, GluA2/3 and PSD95 were analyzed after incubation with CHX for 0, 1, 2, 4 and 6 hours by Western blot. In some experiments, neurons were treated with CHX for 4 h in the presence of DMSO as a vehicle control, proteasome inhibitor lactacystin (10 µM, Sigma-Aldrich) or lysosomal inhibitor bafilomycin A1 (5 nM, Sigma-Aldrich) was used to block specific degradation pathways.

### Co-immunoprecipitation assay

Co-immunoprecipitation (IP) assays were performed using the Pierce crosslink magnetic IP/co-IP kit (Fisher) according to the manufacturer's instructions. Briefly, 2.0 µg of antibodies to LRP1 (made in house), GluA1 (Millipore) and PSD95 (Cell Signaling) were incubated with Protein A/G Magnetic Beads, and then treated with DSS (disuccinimidyl suberate) Crosslinker for 30 minutes. Neurons were lysed at 4°C with Lysis Buffer (0.025 M Tris, 0.15 M NaCl, 0.001 M EDTA, 1% NP-40, 5% glycerol, pH 7.4). Equal protein aliquots (1.0 mg) were immunoprecipitated with the antibody-crosslinked beads for 1 hour at room temperature on a rotator. The precipitates were rinsed with wash buffer and ultrapure water, and eluted by elution buffer. The supernatants were saved and analyzed by Western blot.

### Cell surface biotinylation assay

Cell surface proteins were biotinylated as described [24]. Briefly, neurons were washed twice with ice-cold PBS and cell surface proteins were biotinylated by incubation with 1 mg/ml of EZ-Link Sulfo-NHS-SS-Biotin (Fisher, Pittsburgh, PA) for 30 min at 4°C. Neurons were washed 3 times and lysed in RIPA Buffer (0.15 mM NaCl/0.05 mM Tris-HCl, pH 7.2/1% Triton X-100/1% sodium deoxycholate/0.1% SDS). The cell lysates were centrifuged at 10,000×g for 15 min at 4°C. Biotinylated surface proteins were isolated by immune-precipitating with Streptavidin magnetic beads (Fisher) for 1 h at 4°C, rinsed three times and dissociated in SDS sample buffer. The eluted samples were collected and analyzed by immunoblotting. Some of the cell lysates were analyzed for total GluA1 and GluA2/3 levels, and the ratios of surface/total GluA1 or GluA2/3 levels were calculated.

### Intracellular Ca^2+^ assay

Intracellular Ca^2+^ assay was performed as described previously [25]. Intracellular Ca^2+^ concentrations ([Ca^2+^]i) were determined using microplate reader measurements of fluorescence intensity from cells loaded with the Ca^2+^-sensitive, high affinity fluorescent dye, fluo-4 AM (Invitrogen; Ex = 485±20 nm; Em = 530±20 nm). Neurons were washed in 20 mM HBSS (130 mM NaCl, 5.4 mM KCl, 1.8 mM CaCl_2_, 0.8 mM MgCl_2_, and 5.5 mM glucose at pH 7.4), then incubated with 4.5 µM Fluo-4 AM dye at 37°C for 1 h in dark and washed three times in HBSS to remove extracellular Fluo-4 AM dye. The fluorescence intensity was measured from four locations within each well with the excitation and emission wavelengths set at 494 and 535 nm, respectively. To suppress the activity of the voltage-sensitive Na^+^ channels, voltage-sensitive Ca^2+^ channels, and NMDA receptor channels, neurons were pre-incubated with HBSS containing an antagonist mixture containing 10 µM tetrodotoxin, 50 µM 2-amino-5-phosphonopentanoic acid (APV), and 100 µM nifedipine (all from Sigma) [26]. After recording the basal fluorescence intensity, 30 µM AMPA was added to the cultured medium to stimulate the AMPARs and then fluorescence intensities were measured again. The fold-change in fluorescence was calculated by dividing the final reading of each well by its basal reading.

### Neurite outgrowth and filopodia quantification

Primary cortical neurons were cultured for 8 DIV, infected by lentivirus for 4 days, fixed and stained with mouse anti-MAP2 antibody (1∶1000, Sigma). The total outgrowth and mean process length of more than 25 of neurons from 4 independent cultures were analyzed with the METAMORH software (Universal Imaging Corporation, Downington, PA, USA). To determine filopodia number, fixed cells were imaged using Zeiss confocal microscope with a 63×/1.4NA oil immersion objective. Images were then imported to MetaMorph software. The presence of filopodia was normalized by dividing the total number of filopodia by the cell perimeter (filopodia per µm) [27]. More than 20 of neurons from 4 independent cultures were analyzed. Since lentivirus carrying GFP vector was infected in more than 90% of neurons under the same condition, we assumed that exogenous GluA1 was also expressed to the same extent. Data were analyzed by one-way ANOVA Tukey test.

### Statistical Analysis

All quantified data exclude neurite outgrowth and filopodia quantification represent an average of triplicate samples. Data are presented as mean ± SD or SEM, and analyzed by Prism 4 software. Statistical significance was determined by Student's t-test or one-way ANOVA Tukey test, and *p*<0.05 was considered significant.

## Results

### LRP1-knockdown leads to decreased level of GluA1 in primary neurons

**Figure 1:**
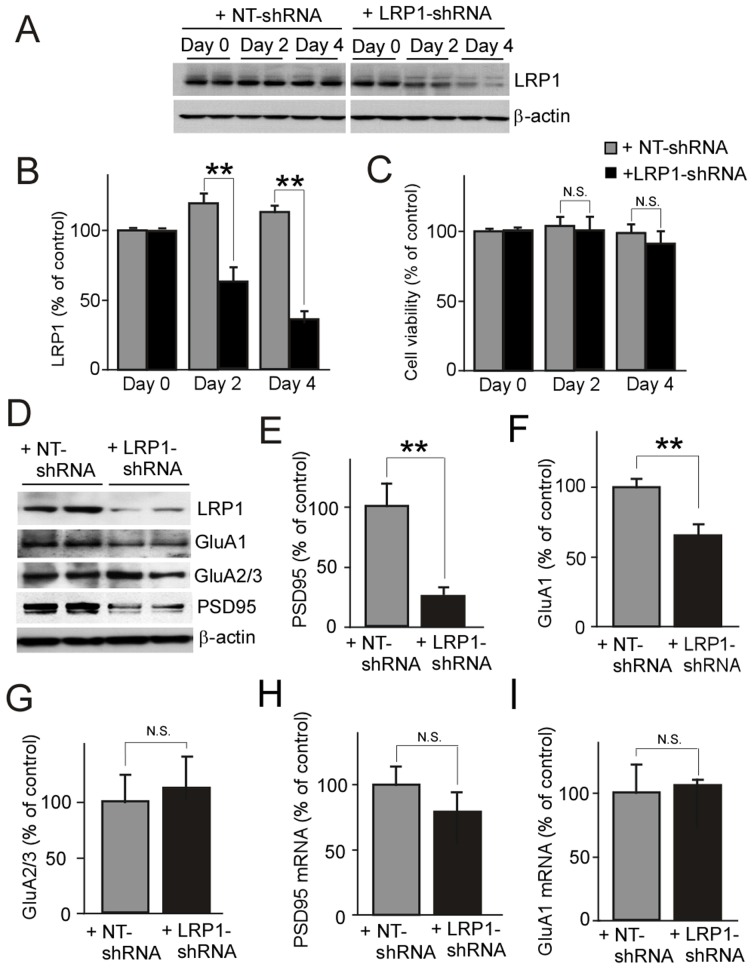
LRP1 knockdown decreases the expression levels of GluA1 in neurons. Primary cortical neurons cultured from C57Bl/6 mice were infected with lentivirus carrying LRP1-shRNA or control NT-shRNA on day 8 *in vitro* (DIV) and then harvested after 2 or 4 days of infection. The expression level of LRP1 in neurons was detected by Western blot (***A***), and densitometrically quantified (***B***). (***C***) The cell viability of neurons was assessed by MTT assay at 2 or 4 days following infection. In LRP1-knockdown neurons, the expression levels of PSD95 (***D***, ***E***), GluA1 (***D***, ***F***), and GluA2/3 (***D***, ***G***) at 4 days post-infection were detected by Western blot and densitometrically quantified. In addition, the mRNA levels of PSD95 (***H***) and GluA1 (***I***) were also analyzed by quantitative real-time PCR. The data are plotted as mean ± SD (n = 3). N.S., Not significant; **, p<0.01.

To investigate potential interaction between LRP1 and AMPARs, we first optimized the method for LRP1-knockdown without affecting cell-viability. When the expression level of LRP1 was analyzed at 2 and 4 days following infection with lentivirus carrying LRP1-shRNA in primary cultures of mouse cortical neurons, we found that LRP1 expression was more efficiently suppressed at day 4 compared to day 2 of knockdown (Figs. 1A and B) with no significant effects on cell viability (Fig. 1C). Since the cell viability started to decrease after infection with LRP1-shRNA for 4 days, LRP1-knockdown neurons were analyzed at day 4 of infection in all following experiments. To assess how LRP1 modulates postsynaptic proteins, we analyzed the expression levels of GluA1, GluA2/3, and PSD95. Western blot analysis revealed that suppression of LRP1 leads to a significant decrease in the levels of PSD95 (Figs. 1D and E) and GluA1 (Figs. 1D and F), whereas no significant changes were detected for GluA2/3 (Figs. 1D and G). To investigate if the decreases in PSD95 and GluA1 were due to transcriptional changes in LRP1-knockdown neurons, quantitative real-time PCR was employed to measure the mRNA levels of PSD95 and GluA1. No significant differences were detected in transcript levels among neurons with or without LRP1 knockdown for PSD95 (Fig. 1H) or GluA1 (Fig. 1I), suggesting that LRP1 regulates these synaptic proteins at the post-transcriptional levels.

### LRP1 forms complexes with GluA1 and controls its stability

**Figure 2:**
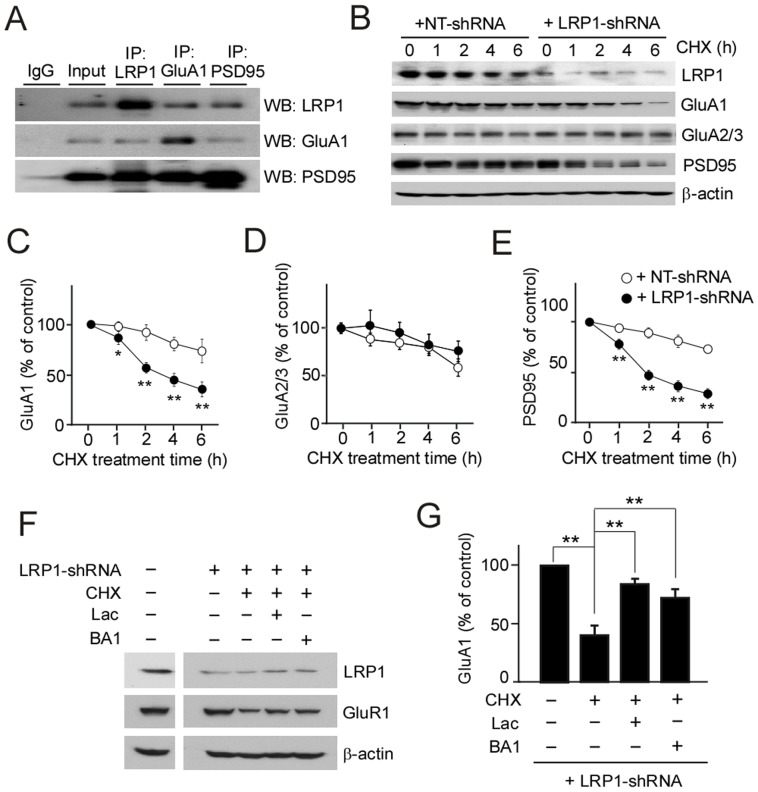
LRP1 interacts with GluA1 and regulates its turnover in neurons. (***A***) Brain lysates from wild-type mice were immune-precipitated using specific antibodies against LRP1, GluA1, GluA2/3 or PSD95, and their interactions were examined by Western blot (***B–E***). After infection with control NT-shRNA or LRP1-shRNA, control and LRP1-knockdown neurons were treated with cycloheximide (CHX), and the levels of GluA1 (***C***), GluA2/3 (***D***) and PSD95 (***E***) were analyzed by Western blot at different time points. (***F***) LRP1-knockdown neurons were treated with DMSO (control), proteasomal inhibitor lactacystin (Lac; 10 µM) or lysosomal inhibitor bafilomycin A1 (BA1; 5 nM) in addition to CHX. (***G***) GluA1 and PSD95 levels were analyzed by Western blot, and densitometrically quantified. The data are plotted as mean ± SD (n = 3). *, p<0.05; **, p<0.01.

Given that LRP1 forms complexes with PSD95 [8], which is present in the postsynaptic complexes with GluA1 [28], we sought to examine potential interaction between LRP1 and GluA1. Consistent with previous findings, we found that PSD95 co-immunoprecipitates with both LRP1 and GluA1 (Fig. 2A). Moreover, LRP1 and GluA1 also co-immunoprecipitate with each other in mouse brains. These results suggest that LRP1 exists in the same postsynaptic complexes with GluA1 in neurons. As LRP1 is a rapid endocytosis and recycling receptor [29], [30], [31], LRP1 might regulate the endocytic trafficking and turnover of other postsynaptic proteins.

To address the possibility that LRP1 might regulate the stability/turnover of GluA1 in neurons, we performed protein stability assays by incubating neurons with CHX to block protein synthesis. We found that in the presence of CHX, the levels of GluA1, GluA2/3 and PSD95 in neurons were decreased in a time-dependent manner when assessed by Western blot (Fig. 2B). In LRP1-suppressed neurons, we found that the disappearance of GluA1 as well as PSD95 was significantly accelerated compared with control neurons (Figs. 2C and E), while the turnover rates of GluA2/3 were not changed (Fig. 2D). To assess the pathways involved in the degradation of GluA1 in neurons, a proteasome inhibitor lactacystin and a lysosome inhibitor bafilomycin A1 were employed, because proteasomal [32] and lysosomal pathway [33] both contribute to the degradation of GluA1 [34]. While LRP1-knockdown reduced GluA1 levels, lactacystin and bafilomycin A1 reversed these effects (Figs. 2F and G). These results indicate that LRP1 controls the turnover of GluA1, but not GluA2/3, in a manner that depends on the functions of both proteasomes and lysosomes.

### LRP1-knockdown decreases surface GluA1 level and suppresses GluA1 phosphorylation in neurons

**Figure 3:**
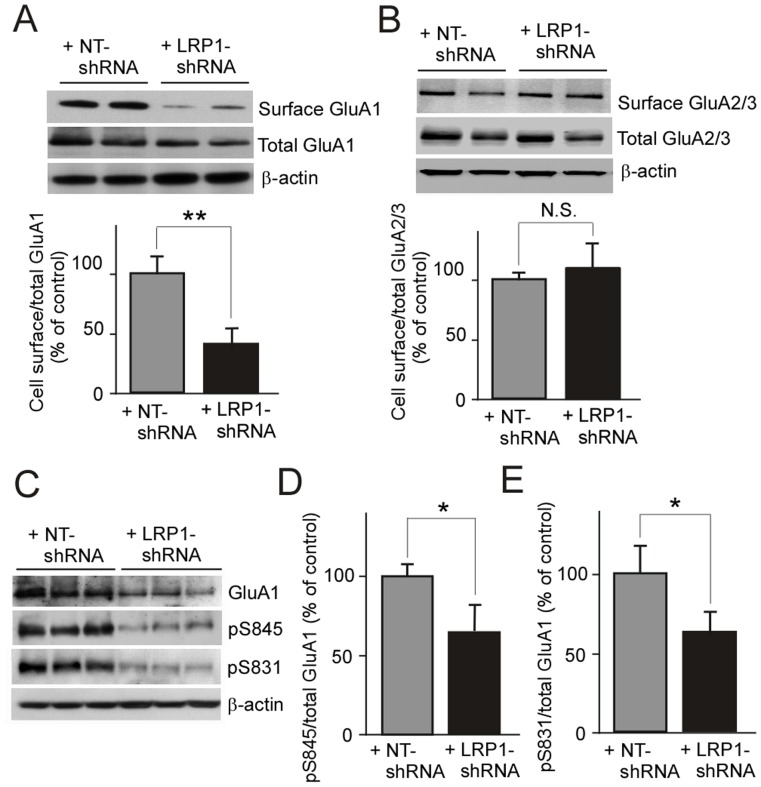
LRP1-knockdown disturbs the trafficking of GluA1 to the cell surface and suppresses GluA1 phosphorylation in neurons. Primary mouse cortical neurons were infected with lentivirus carrying LRP1-shRNA or NT-shRNA for 4 days. Cell surface proteins were labeled with biotin in live neurons, and the cell lysates were precipitated with streptavidin beads. (***A, B***) The precipitates and total cell lysates were examined by Western blot to detect cell surface GluA1 and total GluA1, respectively. The ratio of surface GluA1 versus total GluA1 was quantified (***A***). Similarly, ratio of surface GluA2/3 versus total GluA2/3 was analyzed (***B***). (***C***) In control and LRP1-knockdown neurons, the expression of total GluA1 and phosphorylated GluA1 (pSer-845 and pSer-831) were analyzed by Western blot. The phosphorylation at Ser-845 (***D***) and Ser-831(***E***) sites of GluA1 versus total GluA1 were quantified. The data are plotted as mean ± SD (n = 3). N.S., not significant; *, p<0.05; **, p<0.01.

To assess the effect of LRP1 suppression on the cellular distribution of GluA1, we measured cell surface GluA1 level by cell surface biotinylation assay. Control and LRP1-suppressed neurons were labeled with biotin at 4°C for 30 min. Subsequently, biotinylated cell surface proteins in the cell lysates were isolated by streptavidin beads and cell surface GluA1 level was quantified by Western blot (Fig. 3A). When cell surface level of GluA1 was normalized to the total amount of GluA1 in the cell lysate, we found that cell surface GluA1 decreased to 41.5% of control in LRP1-knockdown neurons (Fig. 3A), while the cell surface levels of GluA2/3 did not change (Fig. 3B). These results indicate that LRP1-deletion facilitates GluA1 internalization and/or suppresses its recycling to the cell surface.

Phosphorylation of GluA1 by PKC or PKA on the cell surface is critical for synaptic plasticity and retention of spatial memory [35]. Furthermore, the phosphorylation affects the trafficking of GluA1 to synapses and/or stabilizes synaptic GluA1 [21], [35], [36]. Thus, we next performed Western blot analysis to explore if LRP1-knockdown affects the phosphorylation of GluA1 in neurons (Fig. 3C). Ser845 and Ser831 are the major phosphorylation sites of GluA1. The results showed that suppression of LRP1 in neurons leads to reduced phosphorylation at Ser845 (Fig. 3D) and Ser831 sites (Fig. 3E). Thus, LRP1-knockdown suppresses phosphorylation of GluA1 by decreasing the cellular distribution of GluA1 to the cell surface, which further disturbs GluA1 stability.

### LRP1-knockdown suppresses GluA1-mediated calcium influx in neurons

**Figure 4:**
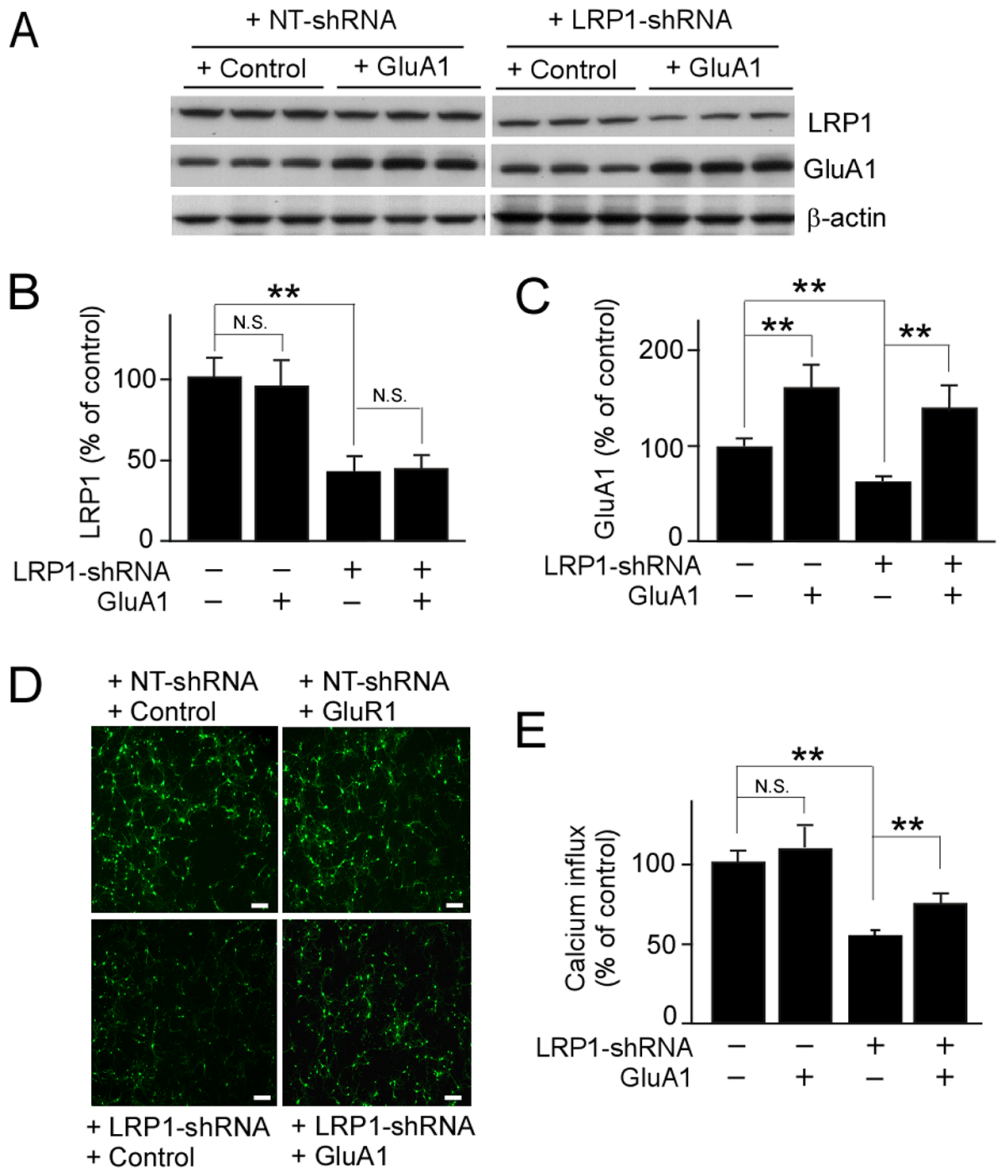
LRP1-knockdown suppresses GluA1-mediated calcium influx in neurons. Primary mouse neurons were first infected with lentivirus carrying control vector or GluA1 plasmid, and then with lentivirus carrying NT-shRNA or LRP1-shRNA (***A***). Expression levels of LRP1 (***B***) and GluA1 (***C***) were detected by Western blot. (***D***) Calcium influx detected with the fluorescence microplate reader using Fluo-4 AM as a fluorescent indicator of intracellular calcium concentration in neurons after stimulation of AMPA in the presence of NMDAR antagonist. The scale bar represents 200 µm. (***E***) Calcium fluorescence intensities were measured with the excitation and emission wavelengths set at 494 and 535 nm, respectively. The data are plotted as mean ± SD (n = 3). N.S., Not significant; **, p<0.01.

Phosphorylation of Ser845 and Ser831 potentiates the ion channel function of GluA1 [37], [38], [39], [40]. Since LRP1-knockdown disturbed the phosphorylation at Ser845 and Ser831 of GluA1 in neurons, we next sought to investigate how LRP1 is involved in GluA1-mediated intracellular calcium influx in neurons. To determine the role of the LRP1-GluA1 pathway, neurons were infected with lentivirus carrying GluA1 plasmid followed by infection with lentivirus carrying control or LRP1-shRNA. Western blot analysis revealed that infection of lentivirus carrying GluA1 plasmid significantly increased GluA1 levels both in control and LRP1-knockdown neurons without affecting LRP1 levels (Figs. 4A–C). To determine the intracellular calcium levels, neurons were incubated with intracellular calcium indicator, Fluo-4 AM dye, and stimulated with AMPA in the presence of NMDAR antagonist APV. The change in the specific fluorescence intensity from baseline after AMPA stimulation was measured as intracellular calcium concentration (Figs. 4D and E). LRP1-knockdown significantly reduced calcium influx in neurons compared with controls. The effects of LRP1-suppression were partially reversed by restoring GluA1 expression through lentivirus-mediated transduction (Figs. 4D and E). Together, these results suggest that LRP1 knockdown impairs synaptic function by decreasing GluA1-mediated calcium influx.

### LRP1-knockdown disturbs GluA1-mediated neurite outgrowth and filopodia formation

**Figure 5:**
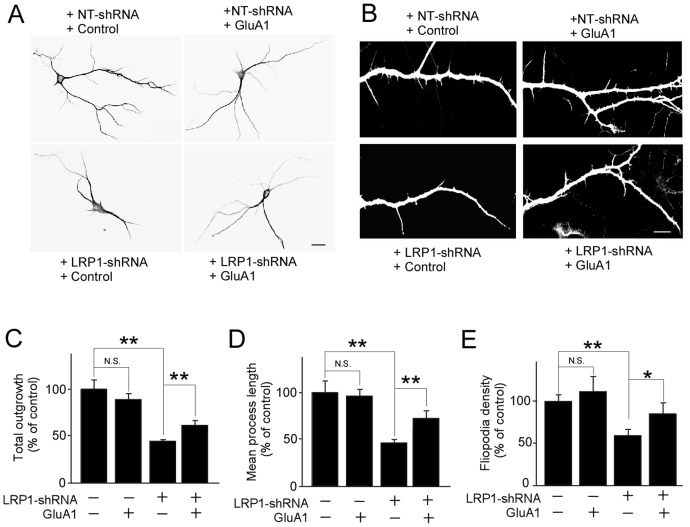
LRP1 regulates GluA1-mediated neurite outgrowth and filopodia formation. Mouse primary neurons were infected with lentivirus carrying control vector or GluA1 cDNA and lentivirus carrying NT-shRNA or LRP1-shRNA. Control and LRP1-suppressed neurons with or without forced GluA1 expression were stained with anti-MAP2 antibody and their neurite outgrowth (***A***; scale bar  = 25 µm) and filopodia formation (***B***; scale bar  = 15 µm) were observed using confocal microscopy. Total outgrowth (***C***), mean process length (***D***) and Filopodia density (***E***) were quantified by MetaMorph software. The data are plotted as mean ± SEM. N.S., Not significant; *, p<0.05; **, p<0.01.

AMPAR activity has been shown to affect synaptic complexity [20], [41]. Thus, we analyzed the effect of LRP1-knockdown on neurite outgrowth and filopodia formation in primary neurons (Figs. 5A and B). When LRP1 level is suppressed by shRNA, total neurite outgrowth (Fig. 5C) and the mean process length (Fig. 5D) were significantly suppressed to 44.8% and 46.9% of the controls, respectively. Furthermore, LRP1-knockdown significantly decreased filopodia density to 52.7% of the control (Fig. 5E). While GluA1 expression did not affect neurite outgrowth and filopodia density in control neurons under our experimental conditions, the effects of LRP1-knockdown were partially, but significantly, restored by rescuing GluA1 expression. These results indicate that LRP1 plays a critical role in maintaining synaptic formation through modulating the function of GluA1.

## Discussion

Increasing evidence has demonstrated a critical role of LRP1 in maintaining synaptic functions and neuronal homeostasis [8], [10], [42], [43]. When the *Lrp1* gene was specifically deleted in differentiated neurons by synapsin promoter-driven Cre recombinase expression, severe behavioral and motor abnormalities were evident at 3–6 months of age [8]. Furthermore, *Lrp1* gene deletion in forebrain neurons in adult mice by αCaMKII-Cre led to compromised brain lipid metabolism and progressive, age-dependent synaptic dysfunction, cognitive impairments, and eventual neurodegeneration [10]. One important pathway by which LRP1 regulates synaptic plasticity depends on functions of glutamate NMDARs receptors at post-synapses [13], [14], [15]. LRP1 interacts with NMDARs through PSD95 and controls its trafficking and degradation [8], [42]. In addition, LRP1 modulates NMDARs function by regulating the binding and function of tissue-type plasminogen activator (tPA) [3], [13]. The LRP1 intracellular domain also regulates NMDAR-mediated signaling pathway [43]. Although effects of LRP1-deletion on NMDARs are well studied, the interaction between LRP1 and AMPARs has received relatively less attention. Thus, in this study, we focused on the dissecting the interaction between LRP1 and the AMPAR subunit GluA1 in mouse primary neurons, and showed that PSD95, LRP1, and GluA1 form immunoprecipitable complexes. PSD95 is a specialized scaffold protein with multiple protein interacting domains that form the backbone of postsynaptic protein complexes at synaptic contact zone [44]. PSD95 recruits and anchors GluA1 to the post-synaptic density [45], [46], [47], [48] through transmembrane AMPAR regulatory proteins (TARPs) [49]. Our results show that the knockdown of LRP1 decreases both GluA1 and PSD95 by accelerating their degradation without affecting their mRNA levels. We further demonstrate that depletion of LRP1 decreases the distribution of GluA1 on the cell surface, which results in suppressed phosphorylation of GluA1 at Ser-845 and Ser-831. While phosphorylation of S845 promotes GluA1 surface expression and affects its stability [50], phosphorylation of Ser831 mediates the delivery of GluA1 to the synapses [36] and increases their single channel conductance [51]. These observations suggest that LRP1 suppresses GluA1 sorting to the degradation pathway and/or facilitates its recycling by influencing its phosphorylation and cell surface distribution. During LTP, AMPARs are recruited to the post-synaptic membrane from non-synaptic pools and interact with scaffold proteins for local concentration. AMPARs can also be targeted to lysosomes for degradation to reduce synaptic density during LTD [19]. Thus, the deletion of neuronal LRP1 likely induces the disturbed LTP and cognitive dysfunction at least partially by suppressing GluA1 trafficking and accelerates its turnover.

AMPARs exist as a tetramer of homologous subunits, GluA1-4 [52]. In hippocampal neurons, GluA1/GluA2 and GluA2/GluA3 heteromers are major AMPAR complexes, whereas approximately 8% of the total is estimated as homomer of GluA1 [53]. While homomeric GluA1 is shown to be calcium-permeable, heteromeric GluA1/GluA2 complex is calcium-impermeable [53]. Synaptic plasticity is differently regulated by the subunit composition of synaptic AMPARs, especially at the level of GluA1 homomers [53], [54], [55], [56], [57], [58]. Interestingly, we found that LRP1-knockdown specifically decreases GluA1, but not GluA2/3, among the AMPAR subunits. Thus, LRP1 selectively regulates the trafficking and function of GluA1 homomers, whose functions include AMPA-evoked calcium influx and synaptic plasticity.

We have also found that LRP1-deletion significantly suppresses neurite outgrowth and filopodia density, effects that were rescued by forced expressing of GluA1. While LRP1 knockdown has been shown to disturb neurite outgrowth by suppressing cholesterol homeostasis [10], our results have demonstrated another potential mechanism by which altered LRP1-GluA1 pathway impacts synaptic formation. Neurite outgrowth is intrinsically linked to cellular calcium levels [59], [60]. As we demonstrated, LRP1-knockdown significantly decreased the intracellular calcium, which was recovered by forced expression of GluA1 in neurons. Thus, the influx of calcium through LRP1-GluA1 likely plays a critical role in outgrowth or retraction of dendrites. In addition, GluA1 expression is associated with greater numbers of filopodia, and an increase in the length and complexity of dendritic arbor, while GluA2 does not alter dendritic complexity but is associated with regulating the length of arbor and the numbers of filopodia [61]. Consistent with these findings, we also observed decreased filopodia in LRP1-knockdown neurons. Thus, our results indicate that LRP1 controls GluA1-mediated synaptic formation, integrity and function, and disturbances of this pathway may be partially involved in synaptic deficits in neurodegenerative diseases. However, LRP1 may regulate neuronal morphology and function through multiple processes as LRP1 mediates endocytosis of a variety of ligands and forms complexes with several cell surface receptors including the NMDA receptors [1], [2], [3].

Increasing evidence indicates that LRP1 is involved in the pathogenesis of Alzheimer's disease (AD), which is the most common form of dementia [62]. As an endocytotic receptor, LRP1 mediates the cellular uptake and subsequent degradation of amyloid-β (Aβ). Since the accumulation, aggregation and deposition of Aβ are imitating events in AD pathogenesis, the disturbance of LRP1-mediated Aβ clearance pathway has been actively studied. In fact, the deletion of neuronal LRP1 also suppresses Aβ clearance, resulting in exacerbated Aβ deposition in brain parenchyma in amyloid AD model mice [12]. In addition, synaptic dysfunction is the earliest feature of AD which is observed prior to Aβ deposition [63], [64], [65]. Detrimental changes in AMPARs are also known to be correlated with synaptic loss in AD brains [66]. Interestingly, brain LRP1 levels are decreased in AD patients and inversely correlate with the age of onset [67]. Further, LRP1 expression in the brain is reduced during normal aging, which is independent of synaptic loss [67]. Thus, the decrease in LRP1 not only disturbs Aβ clearance but also contributes to synapse dysfunction partially by disturbing GluA1 functions during cognitive aging and AD.

In summary, the current study has revealed a novel mechanism by which LRP1 controls synaptic function and structures specifically by regulating GluA1 trafficking, phosphorylation and degradation. These results indicate that LRP1-mediated trafficking of GluA1 to and from the synapses could play a critical role in its steady state levels at synapses to regulate synaptic strength. In addition, it is possible that LRP1 ligands affect the LRP1-GluA1 interaction. Indeed, a major LRP1 ligand apoE is critical in AMPA receptor regulation and LTP [68]. Although further studies are necessary, apoE may strengthen LRP1-GluA1 association at the synapse. Since synaptic dysfunction is a common feature of neurodegenerative diseases including AD, our findings might provide clues as to how the LRP1-GluA1 pathway can be targeted for therapy.
